# A novel small diameter nanotextile arterial graft is associated with surgical feasibility and safety and increased transmural endothelial ingrowth in pig

**DOI:** 10.1186/s12951-022-01268-1

**Published:** 2022-02-08

**Authors:** John Joseph, Vito Domenico Bruno, Nadiah Sulaiman, Alexander Ward, Thomas W. Johnson, Helna Mary Baby, Praveen Kerala Varma, Rajesh Jose, Shantikumar V. Nair, Deepthy Menon, Sarah Jane George, Raimondo Ascione

**Affiliations:** 1 Bristol Heart Institute and Translational Biomedical Research Centre, Faculty of Health Science, University of Bristol, Bristol, BS2 8HW UK; 2 Centre for Nanosciences & Molecular Medicine, Amrita Vishwa Vidyapeetham, Kochi, 682 041 India; 3 Department of Cardiovascular and Thoracic Surgery, Amrita Institute of Medical Sciences & Research Centre, Amrita Vishwa Vidyapeetham, Kochi, 682 041 Kerala India

**Keywords:** Nanotextile, Small diameter vascular grafts, Endothelialisation, Nanofibers, Electrospinning, In-vivo feasibility, Coronary surgery, Vascular surgery, Nanotextile vascular prosthesis, Vascular graft failure, Tissue engineering

## Abstract

Globally, millions of patients are affected by myocardial infarction or lower limb gangrene/amputation due to atherosclerosis. Available surgical treatment based on vein and synthetic grafts provides sub-optimal benefits. We engineered a highly flexible and mechanically robust nanotextile-based vascular graft (NanoGraft) by interweaving nanofibrous threads of poly-L-lactic acid to address the unmet need. The NanoGrafts were rendered impervious with selective fibrin deposition in the micropores by pre-clotting. The pre-clotted NanoGrafts (4 mm diameter) and ePTFE were implanted in a porcine carotid artery replacement model. The fibrin-laden porous milieu facilitated rapid endothelization by the transmural angiogenesis in the NanoGraft. In-vivo patency of NanoGrafts was 100% at 2- and 4-weeks, with no changes over time in lumen size, flow velocities, and minimal foreign-body inflammatory reaction. However, the patency of ePTFE at 2-week was 66% and showed marked infiltration, neointimal thickening, and poor host tissue integration. The study demonstrates the in-vivo feasibility and safety of a thin-layered vascular prosthesis, viz., NanoGraft, and its potential superiority over the commercial ePTFE.

**Graphical Abstract:**

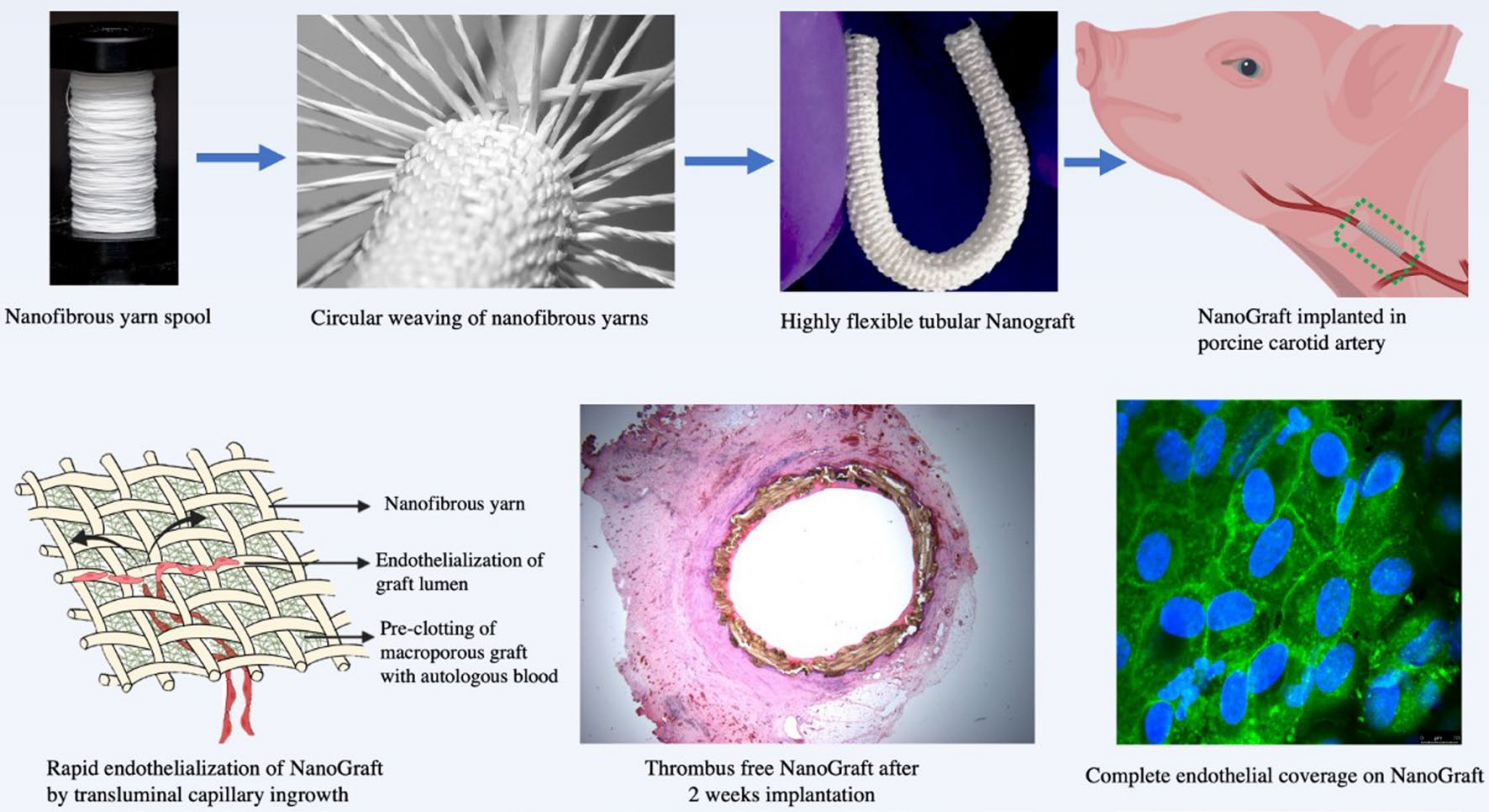

**Supplementary Information:**

The online version contains supplementary material available at 10.1186/s12951-022-01268-1.

## Introduction

Worldwide > 220 million patients are at risk of stroke, myocardial infarction, lower limb gangrene, and amputation due to severe atherosclerosis, a disease mainly affecting arteries with < 6 mm diameter [1]. The established surgical treatments for the most severe forms of coronary and peripheral artery disease include coronary artery bypass grafting (CABG) and lower limb peripheral artery bypass grafting (PABG) using autologous or synthetic small diameter grafts [1–3]. About 80% of the small diameter grafts consist of either autologous saphenous vein grafts (SVGs) or synthetic expanded polytetrafluoroethylene grafts (ePTFE) [4]. However, these grafts encounter 10–15% early thrombosis within 1 month, and 50% occlusion at 1-year due to intimal hyperplasia for CABG-SVGs [4–8] and show similar longevity following PABG for both SVGs and PTFE grafts [9–11]. Hence, there is a critical clinical need for more effective small diameter arterial conduits. Available synthetic ePTFE and polyethylene terephthalate (PET) grafts made from non-degradable materials feature lower patency rates of < 50% at 1-year due to poor endothelialisation, marked intimal thickening, and acute thrombosis [12]. This is triggered by the low flow through the small cross-sectional area and high turbulence at the anastomotic sites. All of these can be attributed mainly to the mismatch in mechanical properties with the native vessel [13, 14]. Different strategies of engineering small vascular grafts have utilized biodegradable polymers, surface modification techniques, tissue engineering, and decellularization of xenografts [15, 16]. None of these approaches have proven success. They fail to address the essential goals for longevity, namely, (i) mechanical properties analogous to the native vessel and (ii) endothelialisation of the internal wall surfaces of the graft to maintain homeostasis and mitigate intimal hyperplasia.

To address these limitations, our group at Amrita (India) developed a novel concept of biodegradable, flexible vascular graft using bundles of nanofibrous threads called yarns [17], which comprises of thousands of individual nanofibers woven together to form nanotextile conduits of predefined small diameter [18]. Woven nanotextiles comprises of interwoven longitudinal and transverse nano-yarns, which collectively enhance the surface area and mechanical property of the vascular graft. Non-woven fibers do not provide the requisite mechanical strength and integrity to serve as a vascular conduit. The use of polymeric nano-yarns offered scalable manufacturing and precise modulation of graft porosity in the micron-scale, which are vital attributes of this patented technology [18]. In this work, this novel vascular graft (NanoGraft) was subjected to preliminary preclinical validation of safety and efficacy at Bristol (UK) under physiological arterial conditions involving in-vitro and in-vivo feasibility testing in a large-animal porcine model of carotid artery replacement. In addition, mechanisms of in-vivo engraftment were studied.

## Materials and methods

The full version of Materials and Methods is shown in the Additional file 1.

### Manufacturing of tubular nanotextiles

Polymeric yarns were produced from poly(L-lactic acid) (PLLA) previously subjected to electrospinning. These were then utilized to make the NanoGraft using a weaving system designed and fabricated in-house [17, 18]. The NanoGraft consisted of a single circumferential yarn that interweaves through multiple longitudinal yarns. The total number of longitudinal yarns used for developing a tightly woven nanotextile was calculated based on the predefined diameter of the conduit. To obtain a 4 mm vascular conduit, a total of 47 longitudinal yarns were interwoven by a single circumferential yarn to attain a tightly woven graft with 330 interweaves per unit area (cm^2^).

### NanoGraft pre-clotting protocol

As the woven NanoGraft was intended for arterial applications, it was subjected to a pre-clotting protocol as described by Sauvage et al. [19]. to prevent leakage. Briefly, the graft was soaked in 10 ml of fresh autologous pig blood and incubated at 37 °C for the next 10 min to allow transmural fibrin formation. Next, it was flushed twice with 10 ml of autologous blood to remove any luminal/mural debris and incubated for 30 s. Finally, the graft was pressurized at 120 mmHg to mimic arterial pressure using heparinized porcine autologous blood (10 ml blood containing 4000 IU Heparin). In this final step, Heparin was used to neutralize any unreacted thrombin.

### Dynamic bench testing of physical properties

The physical properties of the nanotextile conduit were evaluated at the Bristol Heart Institute under arterial pulsatile flow conditions using a bioreactor (TGT DynaGen® Series, USA) primed with fresh heparinized porcine blood (Additional file 1: Fig. S2, Additional file 2: Video 1). To ensure safety, the dynamic testing was conducted at physiological conditions (blood pressure of 120/80 mmHg, ~ 72 cycles/min) and accelerated conditions (blood pressure of 400/350 mmHg, ~ 350 cycles/min) to test mechanical strength. Any oozing/seepage through the grafts was quantified as the volume of leakage per unit area of the graft in unit time.

### Bench testing of biocompatibility at static and dynamic conditions

In-vitro biocompatibility was tested by seeding human umbilical cord vein endothelial cells (HUVECs) described in the Supplemental file. Briefly, we undertook static cell culture over 72 h on small nanotextiles samples aseptically transferred to 96-well plate and seeded with 2 × 10^4^ HUVECs. Next, the samples were washed with phosphate-buffered saline (PBS), fixed with 4% paraformaldehyde (PFA), washed in PBS, dehydrated in gradient ethanol, and imaged using SEM. We then undertook cell cultures on the whole NanoGraft to assess cytotoxicity and amount of engrafted cells. First, HUVECs (10^5^ cells) were seeded in the NanoGraft at static conditions by placing them in a petri dish for 24 h. Next, the NanoGraft was mounted in the bioreactor primed with culture media and run at physiological conditions (pressure 120 mmHg, ~ 72 cycles/min) for additional 24 and 48 h. Then, the NanoGraft was cut into 5 mm segments. Cell presence and viability were tested using the Alamar blue assay, as reported in the Supplemental file. The number of viable cells remaining adherent in each segment of the NanoGraft was obtained from the standard curve. The percentage of adherent cells was calculated by comparing it with static control.

### In-vivo feasibility and mechanisms of engraftment

Upon establishing the in-vitro mechanical safety of the NanoGraft in the dynamic bioreactor study, we undertook the in-vivo feasibility trial in an advanced porcine carotid artery replacement model. The in-vivo feasibility trial was conducted at the Translational Biomedical Research Centre (TBRC) for the large animal at the University of Bristol, Bristol, UK.

The animal procedures were in line with the U.K. Home Office regulations (Animal Act 1986) and were undertaken under a Project Licenses (PPL 30/3064 and PPL: 30/2854) granted by the Home Office after formal review and approval by the University of Bristol Animal Welfare and Ethics Review Body (AWERB). We used female Yorkshire pigs (approx. 60 kg) receiving daily aspirin (300 mg) with food. The procedure was in line with established approaches by our group [20]. General anaesthesia was achieved with IV 0.2 mg/kg morphine and Propofol. Mechanical ventilation was maintained with isoflurane in oxygen/air. Activated clotting time (ACT) > 400 s was maintained with Heparin (10,000–15,000 I.U.) and monitored every 15 min. During the surgical procedure, a continuous infusion of fentanyl five µg/kg/hr was administered along with 0.9% saline (4 ml/kg/hr). After soft vascular clamping 1.5 cm segment of the native carotid artery was excised and 1.8–2.00 cm long grafts, either the pre-clotted Nanograft (n = 3 + 3) or clinical grade ePTFE for control (n = 3), were implanted via end-to-end anastomosis using polypropylene 7–0 sutures (Prolene®, Ethicon, USA). Animals were kept for 2-week followed by termination under general anesthesia. Three additional NanoGrafts were implanted for 4-weeks to prolong the period of observation.

### In-vivo vascular ultrasound doppler

In-vivo vascular ultrasound doppler (USD) was acquired before the implant, 5 min after implant, 2-week for three Nanografts and three ePTFE grafts, and 4-week for three additional NanoGrafts. The USD was acquired under general anesthesia and continuous hemodynamic and surface electrocardiographic monitoring. Lumen size and blood flow velocity were measured and compared within groups and across groups. The inner diameter and intimal thickening of the proximal and distal anastomosis and mid graft portion were determined by 2D Doppler (MySono U6, Samsung, Korea). Blood flow through the implanted graft was determined both qualitatively and quantitatively by using the color Doppler mode.

### Ex-vivo optical coherence tomography—OCT

All the carotid arteries inclusive of all the grafts were explanted at 2 and 4 weeks under general anesthesia at termination and placed in 0.9% saline solution. Next, the lumen area of the excised grafts was assessed using OCT (Zeiss, Germany), with an ex-vivo pullback being performed throughout the length of each specimen.

### Histological evaluation

More detailed methods are presented in the Additional file 1. All the samples were stored at 4 °C in PBS after fixing in 10% Neutral Buffered Saline (NBF) for 24 h. Samples were embedded in paraffin, and sections of 5 µm thickness were assessed for histological findings.

### Hematoxylin and Eosin staining

Samples were stained with Hematoxylin (Sigma Aldrich, USA) for 2 min, then rinsed in distilled water for 3 min. Sections were stained using 0.5% Eosin (Sigma Aldrich, USA), washed for 3 min in tap water, dehydrated in 100% ethanol, rinsed in xylene for 5 min, and imaged using Leica compound microscope (DM500, Germany).

### Verhoeff-Van Gieson Stain for elastin

Sections were stained with Verhoeff’s solution (Sigma, USA) as detailed in the Supplementary file and checked microscopically for elastin fibers. The slides were treated with 5% sodium thiosulfate and washed in running tap water prior to counterstaining with Gieson’s solution for 3–5 min. The specimens were dehydrated quickly with alcohol and mounted with coverslips using DPX mountant (Sigma Aldrich, USA).

### Masson's Trichrome Staining for collagen

The slides were prepared as described in the Additional file 1 and stained in 1% Biebrich scarlet-acid fuchsin solution for 10 min (Sigma, USA). Tissue was differentiated in the phosphomolybdic–phosphotungstic acid solution for 10 min, and the slide transferred into Light green 2% solution, followed by a wash in distilled water.

### Alcian blue (acid mucosubstances)

The slides were stained in a 1% alcian blue solution with 3% acetic acid (Sigma, USA) for 30 min, washed in running tap water for 2 min, and counterstained in the nuclear fast red solution for 5 min.

### Immuno-histofluorescence

Samples were fixed in 10% NBF, washed twice with PBS for the 30 s, and tissue exposed to 200 µl of permeabilization buffer (0.5% Triton-X- 100, Sigma Aldrich, USA) for 10–15 min. Next, additional PBS washing, incubation with 1 mg/ml Bovine Serum Albumin (Sigma Aldrich, USA) for 1 h and exposed to antibody solutions in sequence (primary antibody solution, secondary antibody solution streptavidin Alexa-488, and anti-alpha-smooth muscle-Cy3 antibody) as described in Additional file 1.

## Results

### In-vitro testing of leakage, physical properties, and biocompatibility of the single layered NanoGraft

**Fig. 1:**
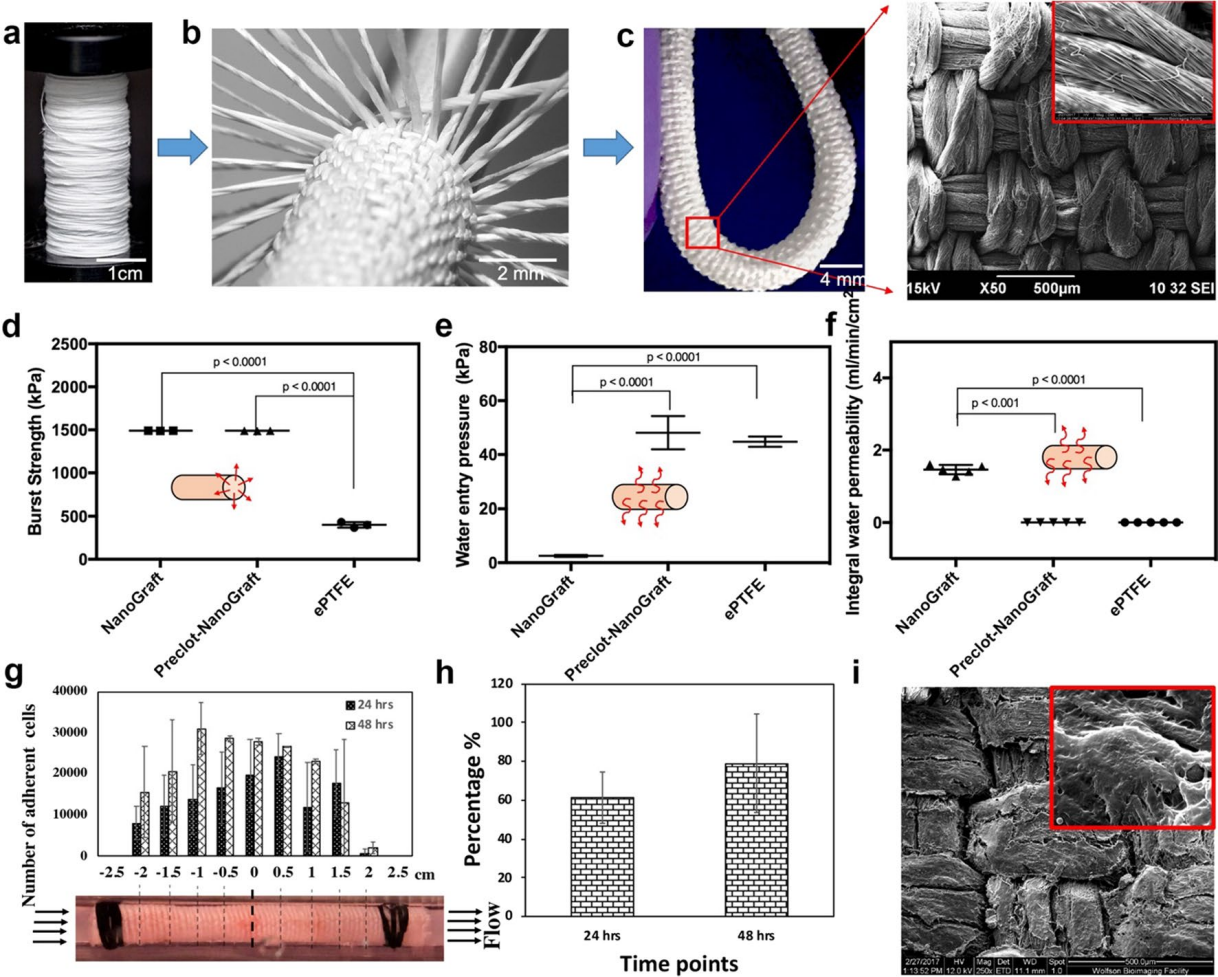
Fabrication and in vitro characterization of the NanoGraft. **a** spool of nanofibrous yarns **b** fabrication of tightly packed woven conduits using multiple electrospun yarns **c** optical image of the NanoGraft (inset shows the SEM micrographs of the nanotextile) **d**, **e** physical properties of NanoGraft compared to the commercial ePTFE graft **g**–**i** adherence of HUVECs on the NanoGraft under dynamic flow conditions **g** Alamar assay showing cell distribution in the NanoGraft **h** the percentage of cells adhered on the NanoGraft under dynamic conditions for 24 and 48 h **i** SEM micrographs of the NanoGraft showing cell coverage

Flexible and kink-resistant nanotextile conduits of 4 mm diameter were developed from polymeric nanofibrous yarns of electrospun PLLA using a modified weaving system as described earlier [18], see Fig. 1a–c. Longitudinal and circumferential yarns were tightly interwoven to create the nanotextile conduit with low porosity, as shown in Fig. 1c (inset). This interwoven structure resulted in a significantly high burst pressure compared to the control ePTFE graft (p < 0.0001) (Fig. 1d). Minimal leakage is an essential requirement for engineered vascular grafts to reach preclinical/clinical applications. To achieve this, the single layered prosthesis was pre-clotted with blood. Bench testing in a dynamic bioreactor (Fig. 1d–f) showed that the non-clotted NanoGraft had marked water leakage when subjected to arterial pulsatile pressures (Fig. 1d). However, the pre-clotted NanoGraft showed significantly reduced water entry pressure and permeability compared to the non-clotted conduit (Fig. 1e–f; both p < 0.0001), which was similar to the water entry pressure and permeability observed for ePTFE control grafts (Fig. 1e–f). Other mechanical properties such as radial stiffness, suture retention, and tensile strength were superior for the NanoGraft (Additional file 1: Figure S1). Thus, despite being single layered, the NanoGraft could satisfy all the vital attributes for a vascular arterial graft.

One key aspect of the in-vitro validation of the Nanograft before in-vivo testing was to assess its biocompatibility under arterial dynamic conditions. Specifically, it was important to assess the endothelialisation potential of this nanotextile conduit. The Alamar blue assay demonstrated the presence of a large number of viable endothelial cells adherent to the NanoGraft and distributed uniformly at 24 and 48 h throughout its length, with the exception at both ends where the cannula was inserted to secure the graft to the flow circuit (Fig. 1g–h). Noticeably, the number of cells increased from 24 to 48 h, indicating cell expansion/proliferation (Fig. 1h). This experiment confirmed that the NanoGraft permitted rapid endothelial cell attachment ((Fig. 1g) and its firm engraftment, given the minimal cell detachment under dynamic arterial conditions (Fig. 1i). This biocompatibility might be due to the superhydrophilicity of the material used, which allows for faster endothelial adhesion, thereby promoting proliferation [21–23].

### In-vivo feasibility testing

**Fig. 2:**
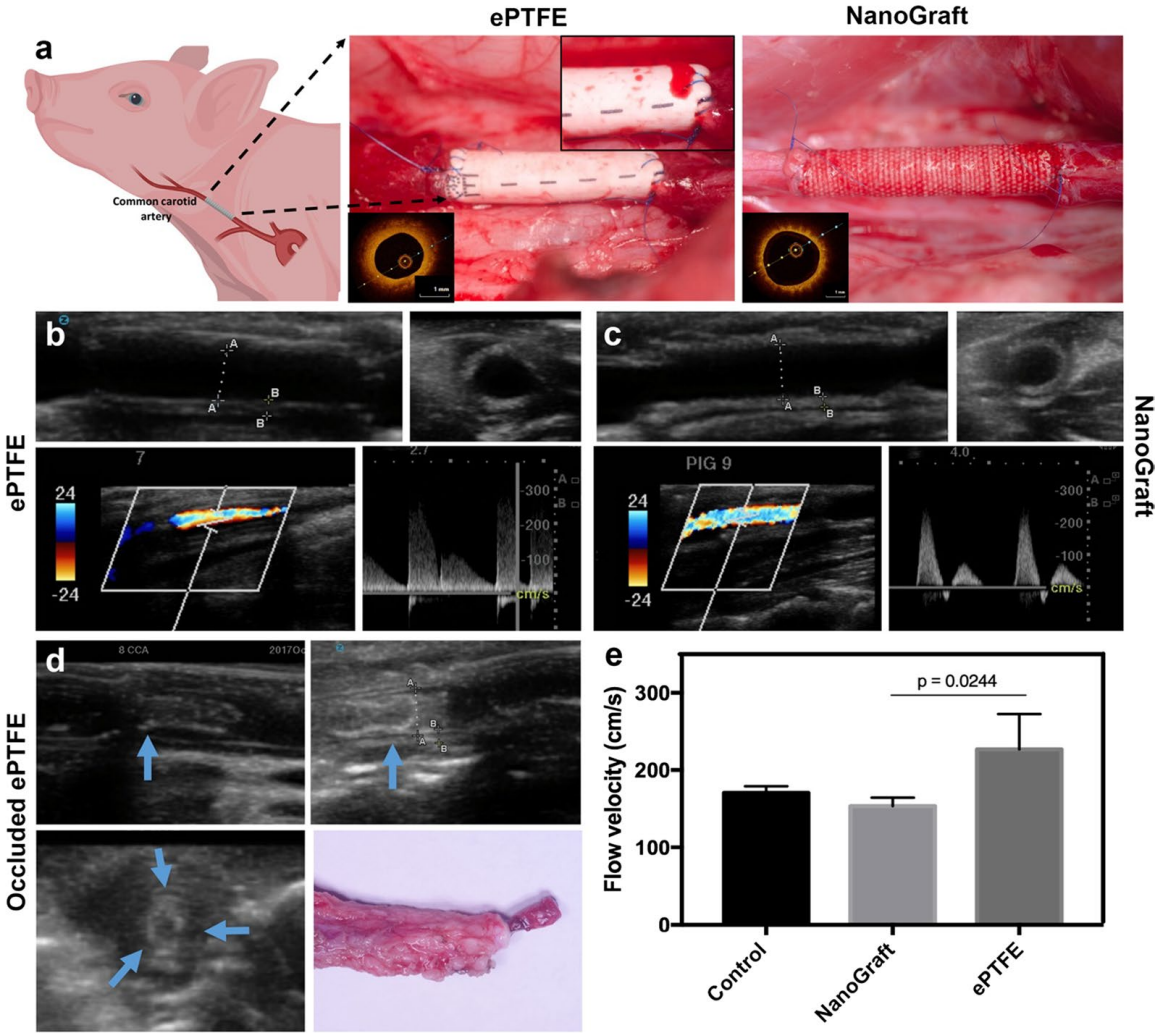
In-vivo implantation and imaging assessment of ePTFE and NanoGraft grafts: **a** direct representative picture of the ePTFE graft with oozing from suture line and NanoGraft with no oozing. Inset shows the OCT image of the patent grafts. Representative 2D Doppler of the longitudinal and cross-sectional view of **b** ePTFE and **c** NanoGrafts (distance A- 3.8 mm, B-0.7 mm), with the corresponding qualitative and quantitative evaluation of blood flow through the grafts (bottom in **b** and **c**). **d** 2D Doppler of the longitudinal and cross-sectional view of the occluded ePTFE grafts (blue arrows depict the regions with adherent clots) and its corresponding optical image. **e** Quantitative evaluation of blood flow through the patent synthetic grafts before and after implantation

Having validated in-vitro the physical properties and biocompatibility of the NanoGraft, we then undertook an in-vivo feasibility trial of pre-clotted NanoGrafts, using clinical-grade ePTFE grafts as control for the 2-week time point. The range of internal diameter of the NanoGrafts was 3.6–3.9 mm. Both NanoGraft and ePTFE grafts exhibited good surgical suturability and handling properties during anastomosis and had a similar vascular cross-clamping time of 12–15 min (Fig. 2a). Additionally, NanoGraft exhibited no signs of fraying at the edges during anastomosis (Additional file 3: Video 2). However, ePTFE grafts showed significant suture line oozing after the restoration of arterial blood flow through the graft (Additional file 1: Figure S2 and Additional file 4: Video 3), which was not observed with NanoGrafts (Additional file 5: Video 4). The anastomotic oozing of the ePTFE grafts resolved 7–10 min after reversing the effect of Heparin with protamine sulfate. All intended procedures were completed successfully and reached the predefined termination point, with no animals being excluded.

### In-vivo vascular Doppler and ex-vivo OCT imaging

USD results showed that the synthetic grafts had an average wall thickness of 400–500 µm, similar to the native carotid artery. All the grafts were patent soon after the surgical implantation. Late graft patency, lumen size, and blood flow velocity are shown in Fig. 2b, c using 2D imaging (longitudinal and transverse) as well as by qualitative and quantitative assessment of blood flow. In-vivo USD imaging just before termination confirmed 100% patency of the NanoGraft at 2-weeks (3/3) (Additional file 6: Video 5) and 4-weeks (3/3), compared to 67% (2/3) for ePTFE control at 2-weeks. One ePTFE graft was occluded at this time point, as shown in Fig. 2d. In addition, at 2-weeks, there was a higher blood flow velocity in ePTFE grafts compared to NanoGrafts and native carotid artery controls (p = 0.02; Fig. 2e). Ex-vivo OCT imaging was conducted post-termination, confirming the early patency of the implanted grafts (see representative images in Fig. 2a inset), correlating with the patency data seen with in-vivo ultrasound Doppler (Additional file 7: Video 6).

### Mechanisms of engraftment, inflammatory response, and vascular remodeling

**Fig. 3:**
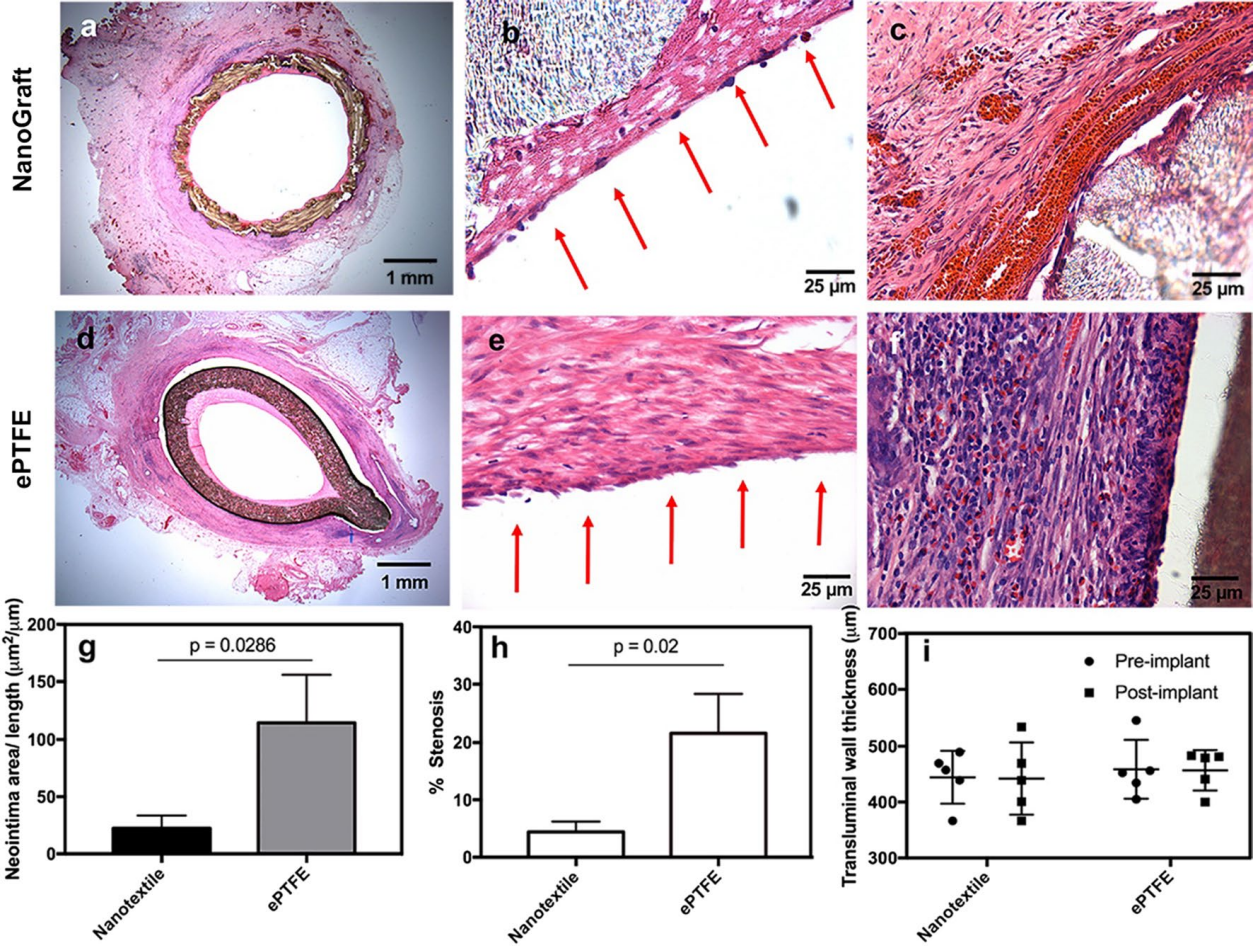
Histological and histomorphometry evaluation of the implanted synthetic grafts after 2 weeks of implantation. H&E staining of the midsection of NanoGraft **a** entire graft Sect. (1.25×) **b** cell lining on the luminal surface of NanoGraft (depicted by red arrows) **c** abluminal surface of the graft with a minimal inflammatory response). **d**–**e** H&E staining of the midsection of ePTFE grafts **d** entire graft Sect. (1.25×) **e** cell lining on the luminal surface of ePTFE (depicted by red arrows) **f** abluminal surface of the graft with high inflammatory cells **g** Neointimal area **h** Percentage of graft stenosis and **i** Transluminal wall thickness, of the implanted synthetic grafts. Statistical significance between the two groups was assessed using a paired t-test. Error bars represent standard deviation. P-value of each comparison is depicted in the plot

Preliminary histology showed no signs of gross thrombi on the luminal surface of the NanoGrafts (Fig. 3a) in contrast to one of the ePTFE grafts, which was entirely occluded by thrombus formation (Fig. 2d and Additional file 4: Figure S3), despite the use of standard platelet inhibition with aspirin. Closer histological evaluation showed neointimal thickening and early endothelialisation in both the grafts (Fig. 3b and e). However, intimal thickening for the NanoGrafts was significantly less than ePTFE grafts (22.39 ± 11.19 vs. 114.71 ± 41.20; p = 0.028; Fig. 3g). No evidence of changes in the transmural wall thickness was observed at 2-weeks for NanoGraft and ePTFE grafts, as shown in Fig. 3i, demonstrating the mechanical integrity of the grafts. The outer diameter of the grafts also did not show any alterations pre and post-implantation, suggesting the absence of late aneurysm or graft rupture. While these early observations are encouraging, this essential aspect needs further validation in long-term in-vivo preclinical studies. Extracellular matrix deposition (ECMD) was observed in the void spaces mainly across the walls of the NanoGraft, between longitudinal and circumferential yarns, with no impact on the lumen size. Conversely, the ePTFE grafts showed a stenotic and non-homogeneous ECMD with neointimal formation, which reduced the lumen by 30–40% compared to baseline (p = 0.02; Fig. 3d, e and h). Inflammatory response observed at the interstitial interface of the NanoGraft was minimal, with excellent tissue integration. (Fig. 3c). In contrast, ePTFE grafts showed a severe inflammatory reaction in the peri-graft region, with a substantial number of neutrophils, lymphocytes and macrophages and no cellular integration (Fig. 3f and Additional file 1: Figure S4).

**Fig. 4:**
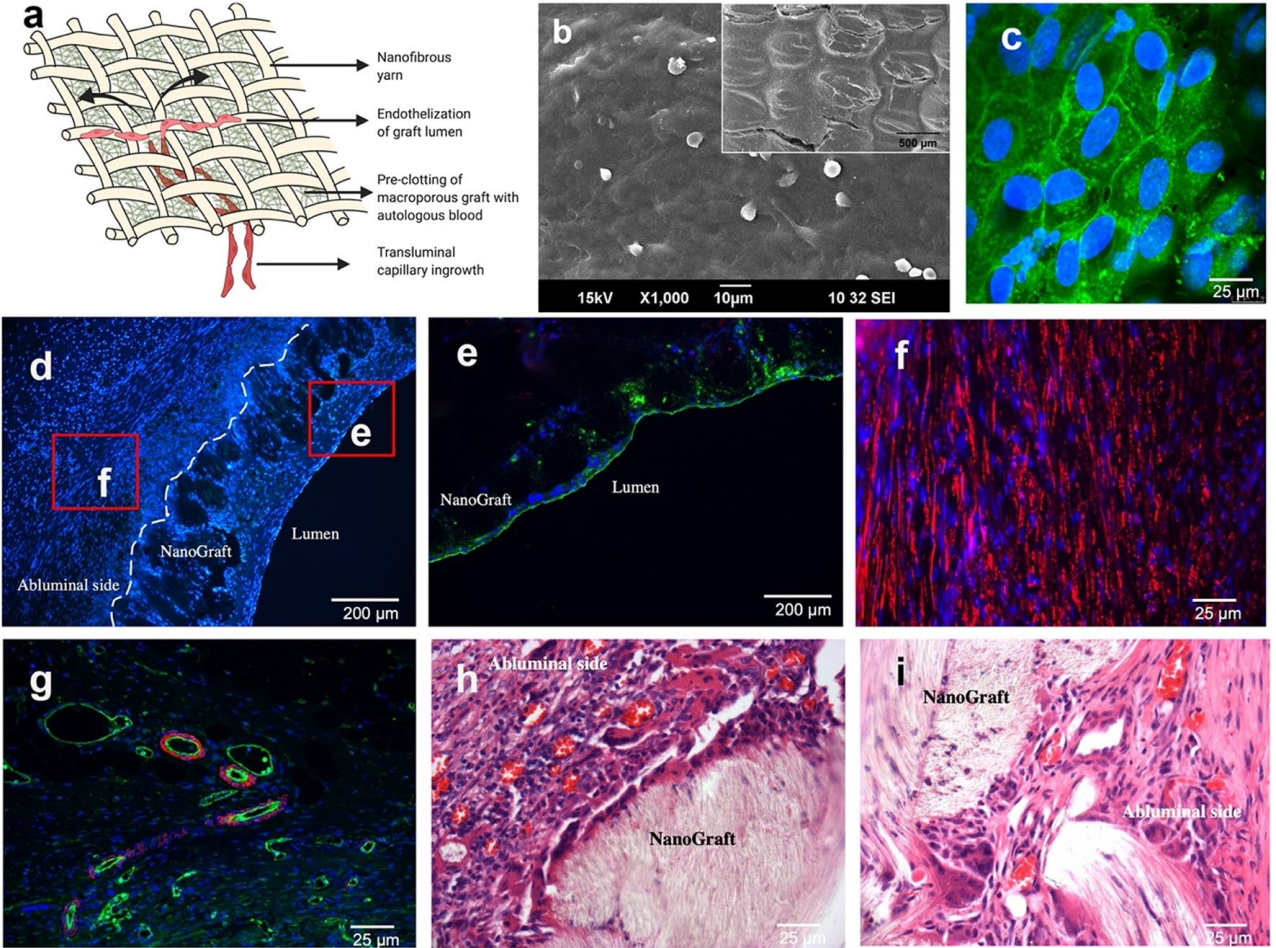
Transmural endothelialisation in biodegradable vascular NanoGraft. **a** Illustration of transmural capillary in-growth through the porous woven structure of the nanotextile graft. **b** SEM micrograph showing endothelial coverage on the entire surface of the graft (inset low magnification). Confocal images of the explanted NanoGraft **c** *en face* staining on the luminal surface of the NanoGraft showing complete endothelial coverage with tight junctions **d** cross-sectional view of vascular graft showing an abundance of infiltrated cells stained for nuclei (DAPI-blue) **e** Immunohistofluorescence staining of the mid-portion of NanoGraft. Tissues stained for nuclei—DAPI (blue), Endothelial cells—Wheat germ agglutinin (green). **f** Presence of circumferentially aligned smooth muscle cells (alpha-smooth muscle actin-red) on the abluminal surface of the graft. **g** Neocapillaries formed at the abluminal side of NanoGraft (400×). H&E staining shows neocapillary in-growth in the **h** abluminal and **i** luminal regions of the porous nanotextile graft

Finally, the intimal thickening observed in the NanoGraft was minimal, with uniform and complete endothelial coverage which can be attributed to its transluminal capillary in-growth as illustrated in Fig. 4a. However, this endothelial lining was patchy in the ePTFE graft*.* This finding was confirmed by the *en face* staining of the endothelial surface of NanoGrafts, as shown in Fig. 4c and Additional file 8: Video 7, and further by SEM micrographs (Fig. 4b). High magnification immunostaining endorsed the formation of tight endothelial junction as shown in Additional file 1: Figure S5. Also, both synthetic grafts showed a large amount of circumferentially oriented smooth muscle cells, as shown in Fig. 4d and f with numerous neo-capillary vessels in the luminal and abluminal regions of the NanoGraft as shown in Fig. 4g–i.

**Fig. 5:**
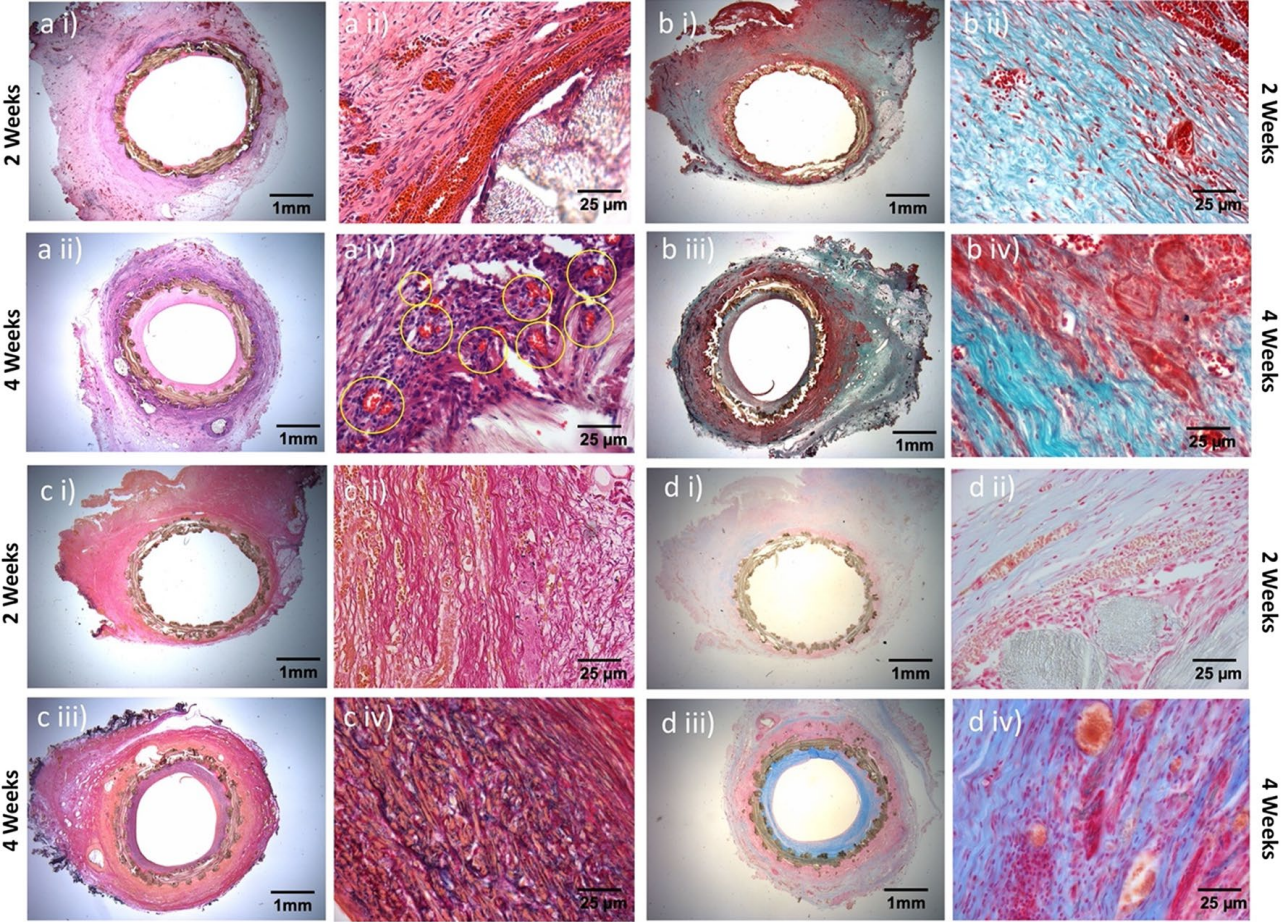
Histological analysis of the NanoGraft showing an increase in neocapillary formation, elastin, collagen, and glycosaminoglycans after 2 and 4 weeks of implantation. **a** H&E staining **b** Masson-Trichrome staining for collagen (bluish-green) **c** Verhoeff-Van Gieson staining for elastin content (blue-black) and **d** Alcian blue stain for mucopolysaccharide (light blue)

A significant transmural neo-capillary formation was observed in NanoGrafts at 4-weeks after surgery, via hematoxylin & eosin staining (Fig. 5a, i–iv). Besides, qualitative and quantitative evaluation of ECMD for collagen, elastin, and mucopolysaccharides revealed an appreciable amount of collagen secretion from the fibroblast/smooth muscle cells, as evident from Masson trichome staining (Fig. 5b, i–iv). Van Gieson stain confirmed elastin deposition at 4-weeks compared to 2-weeks from implantation (Fig. 5c, i–iv). Finally, a higher amount of mucopolysaccharides (Fig. 5d, i–iv) was measured in the NanoGrafts at 4-weeks after surgical implantation, whose absence in synthetic grafts has been reported previously to cause hypercoagulability [24].

## Discussion

Biodegradable grafts have attracted recent interest due to their inherent regenerative potential and ability to resorb over time, eliminating life-long foreign-body inflammatory activation [25–28]. Manufacturing techniques have included electrospinning, casting, and freeze drying [15, 16]. Electrospinning, resulting in nano/micro-scale fibers, has been the most widely used approach in this field so far [27]. Various polymers individually or in combination (PCL, PLLA, PGA, gelatin) and in diverse configurations (aligned, random, hybrid) have been investigated for this purpose [27–29]. Scaffolds fabricated with fibers in this dimension and orientation mimics the extracellular matrix of the native artery, thereby promoting rapid endothelisation, reduced thrombogenicity, cellular infiltration, and regeneration of the polymeric graft [15]. In addition, nano/micro-scale fibers offer better elastic properties, conferring flexibility and manoeuvrability to the vascular graft [29]. However, no biodegradable small arterial grafts have yet been approved by the FDA due to their low burst strength, suture retention, lack of flexibility and potential for kinking, and thereby its inability to meet the essential requirements of the relevant ISO standards 7198:2016 [30, 31]. To address these limitations, we fabricated a vascular graft from bundles of nanofibrous threads called nanoyarns [17] by the textile technique of weaving [18]. This woven nanotextile graft was rendered impervious by a conventional clinical pre-treatment with autologous blood [19], which sealed off the graft interstices by controlled fibrin formation. Excessive heparin containing blood was flushed in the graft lumen to dislodge any surface clots and neutralize unreacted thrombin. Leak test was performed according to the ISO standards and also at physiological pulsatile flow conditions along with high arterial pressure up to 400 mm Hg. Biodegradable grafts fabricated by conventional nanofiber techniques are highly susceptible to mechanical failure at higher arterial pressure, which is common during the post-operative recovery phase, leading to graft failure [32]. In contrast, the NanoGraft revealed high mechanical strength and burst pressure, which can be attributed to the hierarchal arrangement of interwoven nanofibrous yarns along the longitudinal and circumferential directions. Similarly, suture retention of grafts depends greatly on fiber orientation [33]. It is the interwoven pattern utilised in the NanoGraft which conferred it with high suture retention strength compared to the non-woven form [18] and commercial ePTFE grafts (see Additional file 9: Video 8).

In-vivo feasibility studies were conducted at clinical standards, as this is of paramount importance to ascertain the safety and pre-clinical efficacy of the device to enhance its translation potential. Biodegradable grafts must withstand the pressures exerted by pulsatile flow without bursting or experiencing permanent and dangerous deformation through aneurysm or tearing. At the same time, colonization of the graft by inflammatory cells may trigger structural weaknesses. Noticeably, thrombus formation within the grafts can be mitigated if alterations in blood flow velocity across the graft length are minimal, and the luminal graft surface characteristics are non-thrombogenic [34]. In addition, the graft should possess suitable mechanical characteristics to prevent the formation of high shear stress at the anastomotic sites and be of a geometry that does not trigger detrimental flow patterns, as both of these essential factors are associated with graft failure [35]. The formation of micro-thrombi has previously been associated with ePTFE grafts [36]. Also, occlusion of ePTFE grafts has been reported with overall patency of 67% at 1-month when implanted in femoropopliteal position [37] or < 50% when implanted as an arteriovenous graft in the porcine carotid model [38]. Based on this preclinical work and our findings indicating the high rate of graft occlusion at 2-weeks, ePTFE implantation was restricted in our study to 2 weeks only (see Additional file 10: Video 9).

The excellent in-vivo biocompatibility of NanoGraft is an essential factor that may facilitate longevity, which was not observed in ePTFE grafts at the same time point. This observation might be associated with the uniform transmural neo-capillary formation within the walls of the NanoGraft, resembling *vasa-vasorum*, which was absent in ePTFE grafts. The formation of these transmural neo-capillaries may be attributed to the porous nature of the NanoGraft. Similar observations have been made by others related to graft porosity [39, 40]. Additionally, pre-clotting of the NanoGraft would result in a fibrin plug that makes the graft impervious to blood flow. There is ample evidence in literature which suggests enhanced neo-capillary formation in the presence of fibrin [41], supporting our observation. Previous studies have shown endothelial coverage on synthetic graft surfaces to be enhanced by transmural endothelialisation through neo-capillary in-growth from the perivascular region. This process is reported to occur predominantly in grafts with sufficient porosity [39, 40, 42]. Hence, it might be argued that the spontaneous endothelialisation observed in the NanoGraft may be triggered by the presence of fibers in the nanoscale, coupled with the graft porosity. One possible explanation for the observed rapid and uniform endothelial coverage in the NanoGraft is the enhanced adsorption of serum proteins on the superhydrophilic nanotextile [18], which facilitates cellular adhesion and subsequent proliferation in the luminal graft surface. However, a detailed mechanistic study is required to probe and understand the exact mechanisms of engraftment and healing in-situ, wherein the endothelization can also be facilitated or derived from circulating endothelial progenitor cells or migration of endothelial cells from in-situ arteries across anastomotic sites [43].

Synthetics grafts like ePTFE and Dacron® are incapable of spontaneous endothelialisation on the luminal surface of the graft [22, 44]. This is mainly ascribed to the hydrophobic nature of these graft materials, which hinders endothelialisation, and also leads to platelet adhesion and thrombosis. On the contrary, the unique architecture of the nanotextile renders the luminal surface of the NanoGraft superhydrophilic, owing to the aligned nanofibres in the electrospun yarn, which in turn makes the surface antithrombotic.

The deposition of extracellular matrix components such as elastin, collagen and mucopolysaccharides would also provide mechanical integrity during the resorption of NanoGraft. Elastin is expected to increase only after the loss of mechanical integrity of the synthetic graft, observed typically in long-term in-vivo studies. Collagen improves the mechanical resilience [45], while elastin imparts elastic recoil and preserves the structural integrity when exposed to pulsatile flow conditions [46–48]. An increase in collagen and elastin from 2 to 4 weeks shows a promising translational potential of the biodegradable NanoGraft.

## Conclusion

The study confirmed the in-vivo feasibility and safety of the single-layered vascular prosthesis, viz., NanoGraft. Its use appears to be associated with increased transmural in-growth of host cells and neo-capillaries, complete neo-endothelial coverage, patency, and reduced neointimal thickening when compared to ePTFE, although more data is needed to confirm this. Suturability and surgical handling were as good as ePTFE grafts. Physical properties of the NanoGraft were in concurrence with ISO standards, with no dilatation/aneurysm formation observed at 4-week. A pivotal porcine long-term preclinical study is warranted to confirm the safety/efficacy of the proposed graft.

## Supplementary Information

**Additional file 1: **Materials and Methods, **Figure S1.** Mechanical properties of NanoGrafts a) High radial stiffness to resist distortion and compression b) Superior suture retention that demonstrates resistance to wear and tear edges c) Adequate tensile strength to prevent graft rupture. **Figure S2.** ePTFE grafts showed significant suture line oozing after the restoration of arterial blood flow following anastomosis with 7-0 sutures. **Figure S3.** The histopathological finding of the midsection of grafted ePTFE at 2 weeks a) total thrombotic occlusion at the luminal region b and c) higher magnification showing intact fibrin-clot at the graft interface. **Figure S4.** The histopathological finding of the midsection of grafted ePTFE at 2 weeks showing dense infiltration of immune cells in the abluminal section characterised by presence of lymphocytes (white arrows), macrophages (green arrow) and neutrophils (yellow arrows). **Figure S5.** En face immunofluorescence staining of NanoGraft at 2 weeks showed tight endothelial junctions (green -agglutinin on Endothelial, blue- DAPI nucleus).

**Additional file 2:** **Video 1. **Physiological properties of the NanoGraft were evaluated under arterial pulsatile flow conditions using a bioreactor (TGT DynaGen^®^ Series, USA) primed with heparinized porcine blood.

**Additional file 3:** **Video 2.** Nanotextile based graft showed excellent suturability and lack of fraying at the edge.

**Additional file 4:** **Video 3.** ePTFE grafts showed significant suture line oozing after the restoration of arterial blood flow through the graft wall.

**Additional file 5:** **Video 4.** Lack of postoperative suture line bleeding and transmural blood leakage of NanoGraft.

**Additional file 6:** **Video 5.** Percutaneous ultrasound shows the luminal patency and pulsatile blood flow through the Nanograft at 2 weeks.

**Additional file 7:** **Video 6.** Percutaneous ultrasound shows the luminal patency and pulsatile blood flow through the ePTFE graft at 2 weeks.

**Additional file 8:** **Video 7.** Percutaneous ultrasound shows the total occlusion of ePTFE graft at 2 weeks.

**Additional file 9:** **Video 8.** Ex-vivo OCT post-termination confirmed the patency of Nanograft at 2 weeks.

**Additional file 10:** **Video 9.** E*n face* staining of NanoGrafts confirms complete endothelial coverage at two weeks of post-implantation.

## Data Availability

All the raw data used to generate this article is stored on the University of Bristol and Amrita servers with security access. We are happy to make the raw data available if necessary. We have extra aliquots of histology samples available if required. All the pre-clinical research files/daily medical notes for each experiment from surgery, critical care, and maintenance up to the termination are stored under secure access in the Bristol TBRC facility and can be made available to the Editorial Office at any time if requested**.**
