# Correction to: A novel small diameter nanotextile arterial graft is associated with surgical feasibility and safety and increased transmural endothelial ingrowth in pig

**DOI:** 10.1186/s12951-022-01371-3

**Published:** 2022-04-02

**Authors:** John Joseph, Vito Domenico Bruno, Nadiah Sulaiman, Alexander Ward, Thomas W. Johnson, Helna Mary Baby, Shantikumar V. Nair, Deepthy Menon, Sarah Jane George, Raimondo Ascione, Praveen Kerala Varma, Rajesh Jose

**Affiliations:** 1 Bristol Heart Institute and Translational Biomedical Research Centre, Faculty of Health Science, University of Bristol, Bristol, BS2 8HW UK; 2 Centre for Nanosciences and Molecular Medicine, Amrita Vishwa Vidyapeetham, Kochi, 682 041 India; 3 Department of Cardiovascular and Thoracic Surgery, Amrita Institute of Medical Sciences & Research Centre, Amrita Vishwa Vidyapeetham, Kochi, 682041 Kerala India

## Correction to: Journal of Nanobiotechnology (2022) 20:71 https://doi.org/10.1186/s12951-022-01268-1

Following publication of the original article [1], the authors would like to include two new co-authors and revised author’s contributions statement.

1. Dr. Praveen Kerala Varma
  email: varmapk@gmail.com; praveenv21204@aims.amirta.edu
2. Dr. Rajesh Jose
  email: rajeshj21304@aims.amirta.edu

The author group has been updated above and the original article [1] has been corrected.

Authors' contributions

JJ: developed the graft in Amrita, did lab-based work in Bristol, analyzed data and drafted the manuscript; VDB: assisted animal surgery in Bristol and undertook vascular doppler; NS: helped with lab-based work in Bristol and the bioreactor; AW: helped with graft engraftment lab-based work in Bristol; TJ: undertook the ex-vivo optical coherence tomography on explanted grafts in Bristol; HMB: undertook mechanical strength work at Amrita and prepared Fig. 2a, 4a, and graphical abstract; PKV: helped developing the graft in Amrita before studies in Bristol; RJ: helped developing the graft in Amrita before studies in Bristol; SK: helped developing the graft in Amrita and securing previous funding from the Indian Government; DM: helped with developing the graft in Amrita including supervising the Amrita-based bench testing and related interpretation, co-secured the funding for the Commonwealth Scholarship Commission for the Split-site scholarship, co-drafted the manuscript; SJG: supervised the lab-based histology work in Bristol related to graft characterization and interpretation, co-secured the funding for the Commonwealth Scholarship Commission for the Split-site scholarship, co-drafted the manuscript; RA: acted as senior author, conceived and led all the bioreactor and in-vivo work done in Bristol and related interpretation, secured all funding to support bioreactor and in-vivo work in Bristol, co-drafted the manuscript and was corresponding author. All the authors have read and approved the final manuscript.
