# Supplementary material for: A novel small diameter nanotextile arterial graft is associated with surgical feasibility and safety and increased transmural endothelial ingrowth in pig
Source: J Nanobiotechnology. 2022 Feb 8;20:71. doi: 10.1186/s12951-022-01268-1 (PMC8822766; doi:10.1186/s12951-022-01268-1)
Supplement: Supplementary file 1 — Additional file 1: Materials and Methods, Figure S1. Mechanical properties of NanoGrafts a) High radial stiffness to resist distortion and compression b) Superior suture retention that demonstrates resistance to wear and tear edges c) Adequate tensile strength to prevent graft rupture. Figure S2. ePTFE grafts showed significant suture line oozing after the restoration of arterial blood flow following anastomosis with 7-0 sutures. Figure S3. The histopathological finding of the midsection of grafted ePTFE at 2 weeks a) total thrombotic occlusion at the luminal region b and c) higher magnification showing intact fibrin-clot at the graft interface. Figure S4. The histopathological finding of the midsection of grafted ePTFE at 2 weeks showing dense infiltration of immune cells in the abluminal section characterised by presence of lymphocytes (white arrows), macrophages (green arrow) and neutrophils (yellow arrows). Figure S5. En face immunofluorescence staining of NanoGraft at 2 weeks showed tight endothelial junctions (green -agglutinin on Endothelial, blue- DAPI nucleus). [file 12951_2022_1268_MOESM1_ESM.docx]

**A novel small diameter nanotextile arterial graft is associated with surgical feasibility and safety and increased transmural endothelial ingrowth in pig**

John Joseph^1,2^, Domenico Bruno^1^, Nadiah Sulaiman^1^, Alexander Ward^1^, Tom Johnson ^1^, Helna Mary Baby^2^, Shantikumar V Nair^2^, Deepthy Menon^2^*, Sarah Jane George^1^*, Raimondo Ascione^1^*

^1^Bristol Heart Institute, Translational Health Sciences, Bristol Medical School and Translational Biomedical Research Centre, University of Bristol, Bristol BS2 8HW, U.K.

^2^ Centre for Nanosciences & Molecular Medicine, Amrita Vishwa Vidyapeetham, Kochi 682 041, India.

**Corresponding authors:**

Professor Raimondo Ascione, MD, ChM, FRCS,

Bristol Heart Institute, Department of Translational Science,

Bristol Royal Infirmary, level 7, University of Bristol,

Bristol, BS2 8HW, United Kingdom.

Ph: +44 (0) 117 3423286;

Email: [R.Ascione@bristol.ac.uk](mailto:R.Ascione@bristol.ac.uk)

Professor Sarah Jane George, PhD,

Bristol Heart Institute, Department of Translational Science,

Bristol Royal Infirmary, level 7, University of Bristol,

Bristol, BS2 8HW, United Kingdom.

Ph: +44 (0)117 3423154

Email: [S.J.George@bristol.ac.uk](mailto:S.J.George@bristol.ac.uk)

Professor Deepthy Menon, PhD,

Centre for Nanosciences & Molecular Medicine,

Amrita Vishwa Vidyapeetham, Kochi 682 041, India

Ph: +91 484 285 8750

Email: [deepthymenon@aims.amrita.edu](mailto:deepthymenon@aims.amrita.edu)

**Materials and Methods**

*Bench testing of biocompatibility at static and dynamic conditions*

In-vitro biocompatibility was evaluated by seeding human umbilical cord vein endothelial cells (HUVECs) isolated from the human umbilical cord using a standard protocol. Initially, we undertook the static cell culture of small nanotextiles samples aseptically transferred to a 96-well plate. HUVECs (2 x 10^4^ cells) were seeded in each well and incubated in Iscove's modified Dulbecco's medium (IMDM), containing 20% fetal bovine serum (Invitrogen, USA) and endothelial growth supplement (Sigma, USA) at 37^o^C for 72 hours. Next, the samples were washed three times with phosphate-buffered saline (PBS). The cells were fixed using 4% paraformaldehyde (PFA), washed in PBS, dehydrated in gradient ethanol, and imaged using SEM. Then, we undertook static/dynamic cell cultures of the whole engineered NanoGraft to assess its integrity, and the amount of cell engrafted in physiological arterial flow condition (120 mmHg, ~72 cycles/min) with the bioreactor primed with culture media (Endothelial Cell Basal Medium + Supplement mix, Promocell, Germany). For initial static culture, HUVECs (10^5^ cells) were seeded in the NanoGraft (a predefined area with a diameter of 4 mm and length of 4 cm) and allowed to attach to its inner lumen placing in a Petri dish for 24 hours. Next, the NanoGraft was mounted in the bioreactor, and culture media was perfused through the luminal area for additional 24 and 48 hours, respectively. Then, the NanoGraft was cut into 5 mm segments. The Alamar blue assay (Thermo Fischer Scientific, USA) was carried out using the standard protocol to assess cell viability and exclude cytotoxicity. A standard curve was made with increment in cell number (up to 5000 cells/well), and optical density was recorded using a microplate spectrophotometer at 570 nm (Biotex Powerwave X.S., USA). The segments of NanoGraft were incubated in the mixture of Alamar blue and endothelial media in the ratio 92:8 (i.e., 920 µL Alamar blue in 80 µL of complete media) in a 96-well plate for 8 hours.

*In-Vivo feasibility and mechanisms of engraftment*

Upon establishing the in-vitro mechanical safety of the NanoGraft in the dynamic bioreactor study, we undertook the in-vivo feasibility trial in an advanced porcine carotid artery replacement model. The in-vivo pilot trial was conducted at the Translational Biomedical Research Centre (TBRC) for the large animal at the University of Bristol, Bristol, UK.

The animal procedures were in line with the U.K. Home Office regulations (Animal Act 1986) and were undertaken under a Project Licenses (PPL 30/3064 and PPL: 30/2854) granted by the Home Office after formal review and approval by the University of Bristol Animal Welfare and Ethics Review Body (AWERB).

Female Yorkshire pigs (60-70 kg) received Aspirin (300 mg/kg/day) with food from 3 days before surgery till termination. On the day of surgery, animals were sedated with an intramuscular injection of ketamine (15 mg/kg), midazolam (0.2 mg/kg), and azaperone (1 mg/kg). General anesthesia was achieved with IV 0.2 mg/kg morphine and Propofol via auricular vein cannulated with a 22G catheter. After intubation, mechanical ventilation was started, and anesthesia was maintained with isoflurane in oxygen/air. Continuous blood pressure was measured following cannulation of a tarsal artery. A continuous infusion of fentanyl 5 µg/kg/hr was administered along with 0.9% saline (4ml/kg/hr). Activated clotting time (ACT) > 400 seconds was maintained with Heparin (10000-15000 I.U.).

The right carotid artery (C.A.) was exposed through an 8 cm skin incision at the neck. After soft vascular clamping, approx. 1.5cm midsegment of C.A. was excised, and either the pre-clotted Nanograft (n=3+3) or clinical-grade ePTFE for control (n=3) were implanted in a randomized fashion as interposition grafts via end-to-end anastomosis using polypropylene 7-0 sutures (Prolene^®^, Ethicon, USA). The surgical incision was closed using Vicryl (Polygalactin 910, Ethicon, USA) sutures. Upon recovery, pigs were administered with intravenous injection of buprenorphine (0.02 mg/kg) for pain control. The animal was kept for 2-week followed by termination under general anesthesia. Also, three additional NanoGrafts were implanted in 3 more animals (total n=6) and keep for 4-week to prolong the period of observation

*Histological evaluation*

All the samples were stored at 4^0^C in PBS after fixing in 10% Neutral Buffered Saline (NBF) for 24 hours. Samples were embedded in paraffin, and sections of 5µm thickness were assessed for histological findings. In all assays, sections were loaded on treated glass slides (Superfrost™ Plus, Thermo Fischer Scientific, USA), followed by deparaffinization/rehydration with Xylene and gradient ethanol wash (100, 90, 70, and 50%). The following staining techniques were used:

*Hematoxylin and Eosin staining*

Samples were stained with Hematoxylin (Sigma Aldrich, USA) for 2 minutes, followed by a rinse in distilled water for 3 minutes. After that in scot and running tap water for 1 and 3 minutes respectively. The sections were stained using 0.5 % Eosin for 1.2 minutes (Sigma Aldrich, USA) followed by 3 minutes wash in tap water, dehydrated using 100% ethanol, and rinsed twice Xylene for 5 minutes. The samples were mounted with coverslips using DPX mountant (Sigma Aldrich, USA) and imaged using a Leica compound microscope (DM500, Germany).

***Verhoeff-Van Gieson Stain for elastin***

Sections were washed using distilled water and stained with Verhoeff's solution (Sigma, USA) for 1-hour till the color turning black. Samples were then rinsed with tap water followed by differentiation in 2% Ferric Chloride for 1-2 minutes, washed thoroughly in tap water, and checked microscopically for elastin fibers, stained black. The slides were treated with 5% sodium thiosulfate (1 minute) and washed in running tap water before counterstaining with Gieson's solution (3-5 minutes). The specimens were dehydrated quickly with alcohol (95% and two-fold changes of 100% ethanol) and were mounted with coverslips using DPX mountant (Sigma Aldrich, USA).

*Masson's Trichrome Staining for collagen*

The slide was washed in distilled water, stained using Mayer's hematoxylin working solution for 1 minute, followed by rinsing in running warm tap water for 10 minutes and distilled water. Slides were stained in Biebrich scarlet-acid fuchsin solution (1% of Biebrich scarlet and acid fuchsin for 10min, Sigma, USA) and rinsed in distilled water. Tissue was differentiated in the phosphomolybdic-phosphotungstic acid solution for 10min. The slide was then transferred directly into a Light green solution (2% in Light green), followed by a quick wash in distilled water. The specimen was dehydrated very quickly in ethanol (95 and 100 %). The samples were mounted with coverslips using DPX mountant (Sigma Aldrich, USA).

*Immuno-histofluorescence*

For immunohistofluorescence analysis, samples fixed in 10% NBF were thoroughly washed twice with PBS for the 30s. The tissue was permeabilized with 200 µl of permeabilization buffer (0.5 % Triton-X- 100, Sigma Aldrich, USA) for 10-15min at room temperature. The sample was washed twice with PBS and incubated with 1mg/ml Bovine Serum Albumin (Sigma Aldrich, USA) for 1 hour, followed by one PBS wash. An adequate amount of primary antibody solution of Top of Form

Biotinylated Dolichos Biflorus Agglutinin **(**DBA, Vector Laboratories, USA) (1:100) was pipetted and mixed at 4^o^C overnight in blocking buffer (2.5% Goat serum + 0.05% Triton in PBS, Sigma, USA). The specimen was washed twice with PBS, and a sufficient quantity of cocktail containing the secondary antibody solution streptavidin Alexa-488 (Sigma Aldrich, USA) (1:200) and anti-alpha-smooth muscle-Cy3 antibody (Sigma Aldrich, USA) (1:400) in blocking buffer (2.5% Goat serum + 0.05% triton in PBS) was added and incubated at R.T., in the dark, for 2 hours. The tissue sample was washed in PBS and incubated with DAPI (Sigma Aldrich, USA) (1:1000) for 10min followed by PBS wash. The sample was then treated with 0.1% w/v of Sudan black (Sigma Aldrich, USA) in 70% ethanol for 30min. Excess Sudan black was removed and washed with PBS. A drop of antifade mounting media (Sigma Aldrich, USA) was added and covered with coverslip pre-coated with nail polish to seal the side. The slide was imaged using a confocal microscope (Leica SP5 AOBS, Germany). The same protocol was followed for deparaffinized sections (5µm) loaded on treated glass slides (Superfrost™ Plus, Thermo Fischer Scientific, USA). An antigen retrieval step was also adopted for tissue permeabilization, using citrate buffer (at 100^0^C) for 30min.

**Supplemental Material**

All the raw data used to generate this article is stored on the University of Bristol and Amrita servers with security access. We are happy to make the raw data available if necessary. We have extra aliquots of histology samples available if required. In-vivo testing of the NanoGraft was done at a certified MHRA GLP research facility for large animals at the University of Bristol. All the pre-clinical research files/daily medical notes for each experiment from surgery, critical care, and maintenance up to the termination are stored under secure access in the facility and can be made available to the Editorial Office at any time if requested**.**

**Supplementary Figures**

**
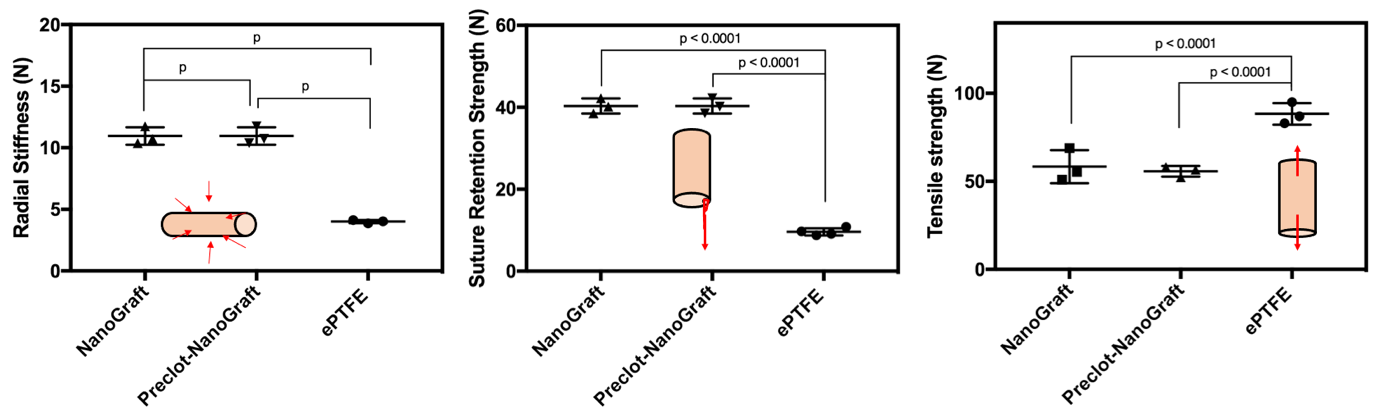
**

**Figure S1.** Mechanical properties of NanoGrafts a) High radial stiffness to resist distortion and compression b) Superior suture retention that demonstrates resistance to wear and tear edges c) Adequate tensile strength to prevent graft rupture

**
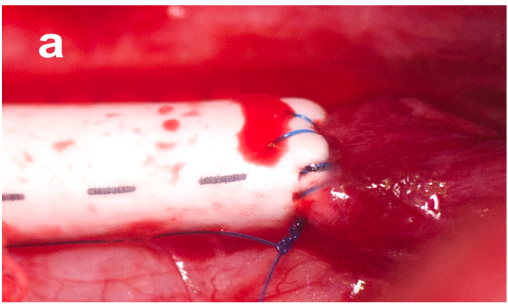
**

**Figure S2.** ePTFE grafts showed significant suture line oozing after the restoration of arterial blood flow following anastomosis with 7-0 sutures

**
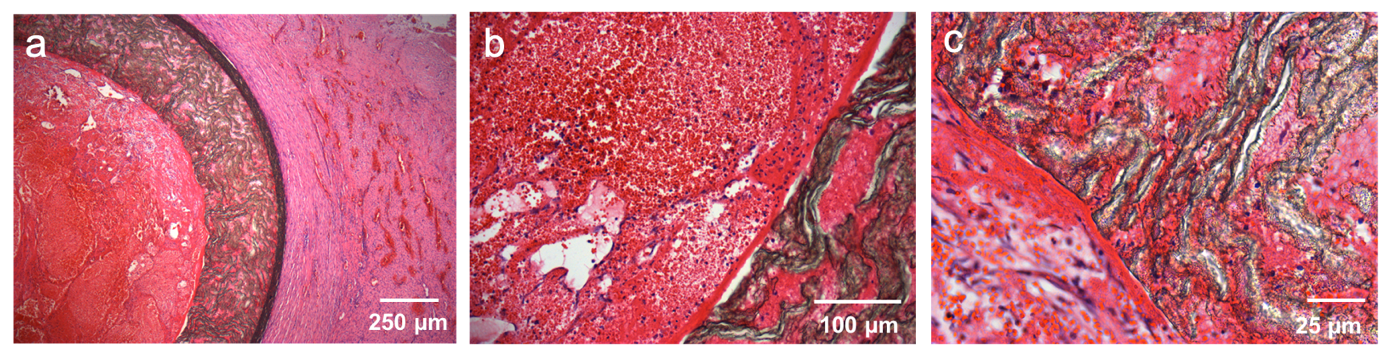
**

**Figure S3.** The histopathological finding of the midsection of grafted ePTFE at two weeks a) total thrombotic occlusion at the luminal region b & c) higher magnification showing intact fibrin-clot at the graft interface

**
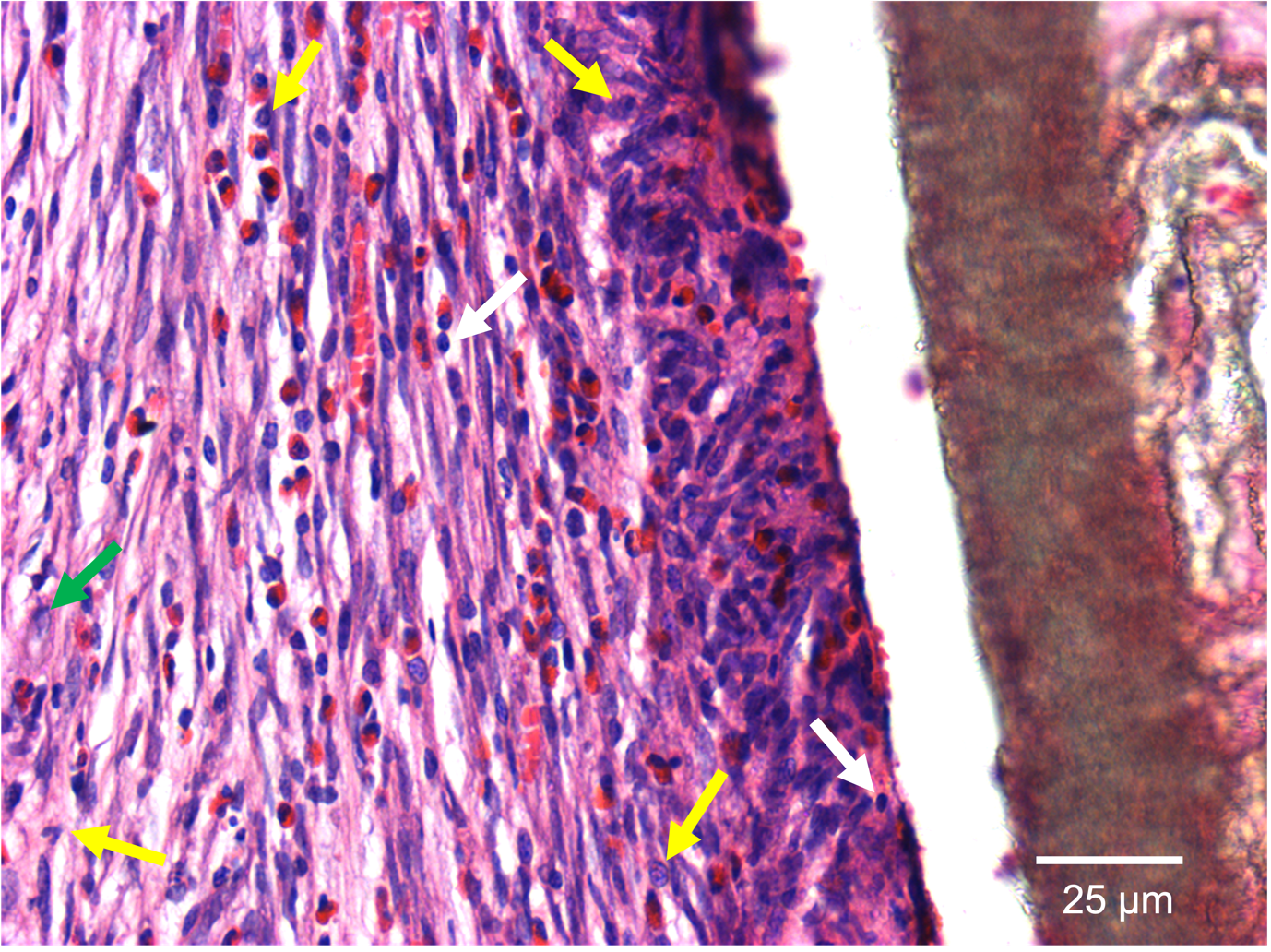
**

**Figure S4.** The histopathological finding of the midsection of grafted ePTFE at two weeks showing dense infiltration of immune cells in the abluminal section characterised by presence of lymphocytes (white arrows), macrophages (green arrow) and neutrophils (yellow arrows).

**
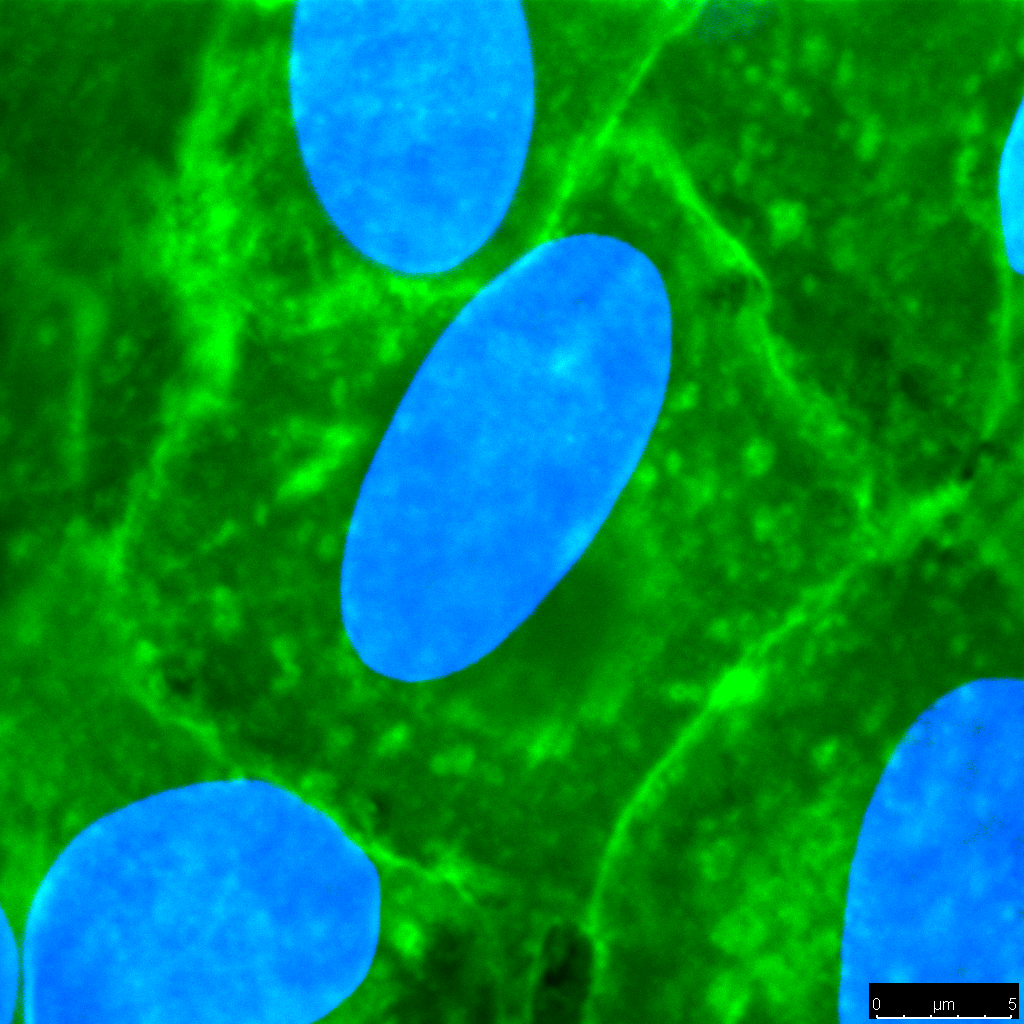
**

**Figure S5** En face immunofluorescence staining of NanoGraft at two weeks showed tight endothelial junctions (green -agglutinin on Endothelial, blue- DAPI nucleus)

**Supplementary Video legends**

**Video 1**

Physiological properties of the NanoGraft were evaluated under arterial pulsatile flow conditions using a bioreactor (TGT DynaGen^®^ Series, USA) primed with heparinized porcine blood.

**Video 2**

Nanotextile based graft showed excellent suturability and lack of fraying at the edge

**Video 3**

ePTFE grafts showed significant suture line oozing after the restoration of arterial blood flow through the graft wall

**Video 4**

Lack of postoperative suture line bleeding and transmural blood leakage of NanoGraft

**Video 5**

Percutaneous ultrasound shows the luminal patency and pulsatile blood flow through the Nanograft at 2 weeks

**Video 6**

Percutaneous ultrasound shows the luminal patency and pulsatile blood flow through the ePTFE graft at 2 weeks

**Video 7**

Percutaneous ultrasound shows the total occlusion of ePTFE graft at 2 weeks

**Video 8**

Ex-vivo OCT post-termination confirmed the patency of Nanograft at 2 weeks

**Video 9**

E*n face* staining of NanoGrafts confirms complete endothelial coverage at two weeks of post-implantation
